# Apigenin inhibits proliferation and induces apoptosis in human multiple myeloma cells through targeting the trinity of CK2, Cdc37 and Hsp90

**DOI:** 10.1186/1476-4598-10-104

**Published:** 2011-08-29

**Authors:** Ming Zhao, Jian Ma, Hai-Yan Zhu, Xu-Hui Zhang, Zhi-Yan Du, Yuan-Ji Xu, Xiao-Dan Yu

**Affiliations:** 1 Department of Stress Medicine, Institute of Basic Medical Sciences, Cognitive and Mental Health Research Center, Beijing, 100850, China; 2 International Medical Center, General Hospital of PLA, Beijing 100853, China; 3 Department of Hematology, General Hospital of PLA, Beijing 100853, China

**Keywords:** Apigenin, CK2, Cdc37, Hsp90, Multiple myeloma (MM)

## Abstract

**Background:**

Multiple myeloma (MM) is a B-cell malignancy that is largely incurable and is characterized by the accumulation of malignant plasma cells in the bone marrow. Apigenin, a common flavonoid, has been reported to suppress proliferation in a wide variety of solid tumors and hematological cancers; however its mechanism is not well understood and its effect on MM cells has not been determined.

**Results:**

In this study, we investigated the effects of apigenin on MM cell lines and on primary MM cells. Cell viability assays demonstrated that apigenin exhibited cytotoxicity against both MM cell lines and primary MM cells but not against normal peripheral blood mononuclear cells. Together, kinase assays, immunoprecipitation and western blot analysis showed that apigenin inhibited CK2 kinase activity, decreased phosphorylation of Cdc37, disassociated the Hsp90/Cdc37/client complex and induced the degradation of multiple kinase clients, including RIP1, Src, Raf-1, Cdk4 and AKT. By depleting these kinases, apigenin suppressed both constitutive and inducible activation of STAT3, ERK, AKT and NF-κB. The treatment also downregulated the expression of the antiapoptotic proteins Mcl-1, Bcl-2, Bcl-xL, XIAP and Survivin, which ultimately induced apoptosis in MM cells. In addition, apigenin had a greater effects in depleting Hsp90 clients when used in combination with the Hsp90 inhibitor geldanamycin and the histone deacetylase inhibitor vorinostat.

**Conclusions:**

Our results suggest that the primary mechanisms by which apigenin kill MM cells is by targeting the trinity of CK2-Cdc37-Hsp90, and this observation reveals the therapeutic potential of apigenin in treating multiple myeloma.

## Background

Multiple myeloma (MM) is a B-cell malignancy characterized by the accumulation of malignant plasma cells in the bone marrow. Despite the use of conventional or high-dose chemotherapy or autologous stem-cell transplantation, tumor cells invariably generate a resistance to the various treatments. Chemoresistance of MM cells remains the primary obstacle in developing a satisfactory treatment [1]. Therefore, to improve outcomes and extend the length of survival, the establishment of more effective treatments that can overcome or circumvent chemoresistance has become a priority.

Casein kinase 2 (CK2) is a ubiquitous cellular serine-threonine kinase with a broad spectrum of substrates. CK2 participates in the regulation of multiple biologic processes and plays an important role in regulating multiple cellular functions, including transcription, translation, signal transduction and metabolism [2,3]. The expression and activity of CK2 are frequently elevated in cancer cells, which provides a growth advantage because its activity counteracts apoptosis and sustains the cell cycle [4,5]. It has been shown that MM cell lines and highly purified malignant plasma cells in patients with MM expressed higher protein and CK2 activity levels than normal plasma cells and B lymphocytes [6]. In this regard, using siRNA to inhibit CK2 activity induced apoptosis and enhanced the cytotoxic effect of melphalan on MM cells. It was proposed that CK2 might play a pivotal role in controlling survival and sensitivity to chemotherapeutics of MM cells [6]. The exact mechanisms governing the pleiotropic activity of CK2 have not been well defined. However, some recent studies have demonstrated that CK2 controls Hsp90 chaperone machinery by phosphorylating a kinase-targeting molecular co-chaperone, Cdc37 [7,8].

Among Hsp90 co-chaperones, Cdc37 is unique because it interacts with a subset of client kinase proteins within Hsp90 complexes and plays a specialized role as a primary partner in kinome maintenance [9]. Cdc37 plays a role in protein kinase quality control not only by protecting nascent polypeptide chains from degradation and by promoting posttranslational maturation [10]. CK2-mediated phosphorylation of Cdc37 on a conserved Ser13 in the N-terminal region is important for efficient binding to client kinases and for recruiting Hsp90 to the kinase-Cdc37 complex [7,8]. Therefore, CK2 activity also depends on Cdc37; there is a positive feedback loop between CK2 and Cdc37 which positively regulates multiple protein kinases [11]. Hsp90 binds to and protects CK2 from self-aggregation and enhances its kinase activity [12]. Strikingly, several crucial anticancer targets, including EGFR, PDGFR, Aurora B, Src, Raf-1, AKT, IKK, Cdc2, Cdk2, Cdk4, and Cdk6 are Cdc37 client kinases http://www.picard.ch/downloads/Cdc37interactors.pdf. Because the function of Hsp90/Cdc37 determines the stability and activity of these kinases, the dependency of the cancer cell kinome on Hsp90/Cdc37 makes the CK2-Cdc37-Hsp90 trinity a promising anti-cancer drug target [13].

Cdc37 is overexpressed in several types of cancers, including multiple myeloma [9]. Previous studies have shown that RNA interference-mediated downregulation of Cdc37 enhances the cytotoxic effects of Hsp90 inhibitors in prostate cancer cells and colon cancer cells by reducing client kinase activity and decreasing survival signaling [14,15]. Treating cells with 4, 5, 6, 7-Tetrabromobenzotriazole (TBB), which is a specific chemical inhibitor of CK2, induces a decline in phosphorylation of Cdc37 and decreases the intracellular levels of Cdc37-dependent protein kinases [8]. However, an evaluation of the strategies of killing cancer cells by inhibiting CK2-dependent phosphorylation of Cdc37 has not been reported.

The flavonoid apigenin is abundant in common fruits and vegetables. Apigenin has gained attention because it has notable anti-inflammatory, antioxidant and anti-carcinogenic properties [16]. Apigenin has been shown to be remarkable in inhibiting growth, arresting cell cycle and inducing apoptosis of human prostate cancer, breast cancer and leukemia [17-21]. Possible mechanisms mediating its anticancer effects include modulation of various kinase activities [17], inactivation of NF-κB [18], inhibition of proteasomal activity [19] and induction of proteasomal degradation of the Her2/neu proteins [20]. As a selective CK2 kinase inhibitor, apigenin has been reported to induce cell death to a greater extent in CK2α-high AML than in CK2α-low AML or normal BM samples [21]. However, the detailed mechanism by which targeting CK2 leads to apoptosis and inactivation of survival signals has not been defined. Given that MM cells also exhibit high CK2 activity, it was of interest to determine the ability of apigenin to kill MM cells. In the present study, we have investigated the effects of apigenin on MM cell lines and purified primary MM cells. We found that apigenin inhibited the proliferation of MM cells, and induced apoptosis of MM cells through the suppression of CK2 kinase and the reduction of Cdc37 phosphorylation. These effects disrupted the Hsp90 chaperone function and downregulated multiple client kinase proteins, and as a consequence, induced apoptosis in MM cells.

## Methods

### Reagents and antibodies

Apigenin, MG132, Geldanamycin and α-tubulin antibody were obtained from Sigma-Aldrich (St Louis, MO, USA), and suberoylanilide hydroxamic acid (SAHA or Vorinostat) was donated by AstraZeneca (Macclesfield, SK, UK). These reagents were dissolved in DMSO (less than 0.1%, v/v). Recombinant human (rh) IL-6 and rhIGF-1 were purchased from PeproTech (Rocky Hill, NJ, USA). Antibodies against phospho-AKT (Ser473), AKT, phospho-ERK (Thr202/Tyr204), ERK, phospho-STAT3 (Tyr705), STAT3, phospho-IκB-α (Ser32), phospho-PDK1, PDK1, phospho-MEK, MEK, phospho-IKK, poly (ADP-ribose) polymerase (PARP), and XIAP were obtained from Cell Signaling Biotechnology (Beverly, MA, USA). Antibodies against Survivin, Mcl-1, IKK and Cdc37 were purchased from Santa Cruz Biotechnology (Santa Cruz, CA, USA). Anti-β-actin, phosphoserine, CK2α antibodies and tetrabromobenzotriazole (TBB) were obtained from Calbiochem (San Diego, CA, USA). Anti-Raf-1, Bcl-2, Bcl-xL and Cdk4 antibodies were purchased from BD Biosciences (San Diego, CA, USA). The anti-Src antibody was purchased from Upstate Biotechnology (Lake Placid, NY, USA). The anti-Hsp90 antibody was obtained from Stressgen Biotechnologies (Victoria, BC, Canada). The anti-RIP1 antibody was purchased from Abcam (Cambridge, MA, USA).

### Cell lines and clinical samples

The human MM cell lines (U266 and RPMI 8226) were obtained from the American Type Culture Collection (ATCC; Rockville, MD) and cultured in RPMI 1640 medium containing 10% heat-inactivated fetal bovine serum (FBS) and 100 U/ml penicillin-streptomycin. The human cervical carcinoma cell line (HeLa) were cultured in DMEM medium with 10% FBS. Bone marrow samples were obtained from patients with MM that underwent treatment at the General Hospital of PLA (Beijing, China), and approval was obtained from the hospital institutional review board for these studies. Informed consent was obtained from all patients in accordance with the Declaration of Helsinki. The CD138^+ ^cells were separated by immunomagnetic bead selection (Miltenyi Biotec GmbH, Bergisch Gladbach, Germany). The purity of isolated CD138-positive plasma cells was approximately 95% as assessed by flow cytometry using phycoerythrin (PE)-conjugated monoclonal CD138 antibodies. To generate peripheral blood mononuclear cells (PBMCs), 5 ml of whole blood was collected from five healthy donors. PBMCs were enriched by density centrifugation over Ficoll-Paque density gradient. The mononuclear cell fraction was collected and washed three times in sterile PBS and was immediately used in the cytotoxicity assays.

### Cell Viability Assay

The cell viability was determined by the 3-(4, 5-dimethylthiazol-2-yl)-5-(3-carboxymethoxyphenyl)- 2-(4-sulfophenyl)-2H-tetrazolium (MTS) assay according to the manufacturer's instructions (CellTiter96 Aqueous Non Radioactive Cell proliferation Assay kit; Promega, Madison, WI, USA). MM cell lines were cultured in complete medium containing the vehicle DMSO (control, less than 0.1%, v/v) or complete medium supplemented with various concentrations of apigenin or TBB for 24 h and 48 h. At the end of the incubation period, 20 μl of the combined MTS/PMS solution was added into each well of the 96-well plate. Following 4 h of incubation at 37°C and 5% CO_2_, absorbance was detected at a wavelength of 490 nm. The results are presented as means ± SD from three independent experiments. Inhibition graphs were plotted using mean values obtained from each concentration relative to control values.

### Cell cycle analysis

Log-phase U266 and RPMI 8226 cells were seeded in 6-well plates and treated with varying dose of apigenin or vehicle DMSO for 24 h. The cells were harvested, washed with PBS and fixed with 70% ethanol containing 1% FBS at -20°C overnight. After an additional washing step, cells were incubated with RNase A (20 μg/ml) at 37°C for 30 min, stained with propidium iodide (PI, 100 μg/ml) for 10 min, and analyzed by flow cytometry.

### Apoptosis assay

Apoptosis was determined with the Annexin-V-FLUOS staining kit (Roche, Indianapolis, IN, USA) according to the manufacturer's instructions. Briefly, the vehicle DMSO control and the apigenin-treated cells were collected by centrifugation and were washed one time with PBS. The cells were subsequently stained with fluorescein and PI for 15 min at room temperature and analyzed by flow cytometry.

### CK2 kinase activity assay

CK2 kinase activity in cell lysates was measured by using the Casein Kinase-2 Assay Kit (Upstate Biotechnologies, Temecula, CA, USA) as described before [21]. Briefly, 20 μg whole-cell lysates were tested in Assay Dilution Buffer I (ADBI) plus with 200 μM substrate peptide (RRRDDDSDDD), 2 μM PKA inhibitor peptide(PKI-[6-22]-NH_2_), and 100 μCi [γ-^32^P] ATP. The reaction mixtures were incubated with agitation for 10 min at 30°C. Reactions were stopped by addition of 40% trichloroacetic acid (TCA). Samples (25 μl) were then transferred onto phosphocellulose filter paper square P81, and the radiolabeled substrate was allowed to bind to the paper for 30 sec. The paper was immersed in 0.75% phosphoric acid and mixed gently on a rotator; followed by washing six times with 0.75% phosphoric acid and one wash with acetone for 1 min. Radioactivity incorporated into the substrate peptide was determined by scintillation counting.

### Immunofluorescence analysis

The vehicle-only control and apigenin-treated cells were fixed for 10 min in PBS containing 4% paraformaldehyde and permeabilized with 0.25% Triton X-100 for 10 min. After washing 3 times with PBS, the cells were immersed in 1% bovine serum albumin (BSA) for 30 min and were incubated with primary anti-CK2α antibody (1:50) overnight at 4°C. After additional washing with PBS, the cells were incubated with secondary antibody conjugated with FITC for 1 h in the dark at room temperature. The cells were examined either by flow cytometry or by fluorescent microscopy at total 1000× magnification under immersion oil using a LSM 510 META ZEISS fluorescent microscope. The fluorescence intensity of CK2α protein was quantified using SoftWoRx Explore 1.2 (Applied Precision, Issaquah, USA).

### RNA interference

Small interfering RNA (siRNA) oligonucleotides were synthesized by GeneChem Co., Ltd (Shanghai, China). The sequence for CK2α was 5'-GAUGACUACCAGCUGGUUCdTdT -3' and the control siRNA sequence was 5'- UUCUCCGAACGUGUCACGUTT-3'. The siRNAs were introduced into HeLa and MM cells by RNAiFect Transfection Reagent or electroporation respectively. HeLa cells were transfected with 40 nM siRNA using the RNAiFect Transfection Reagent (Qiagen, Germany) according to the manufacturer's instructions. Log-phase U266 and RPMI 8226 cells were harvested, washed once and resuspended in serum-free RPMI1640 medium at a concentration of 1 × 10^7^/ml. Control siRNA (75 pM) or CK2α-siRNA (75 pM) was added to 200 μl cell suspension. Next, the mix was transferred directly into a 2 mm gap electroporation cuvette and was electroporated with an Electro Square Porator ECM830 (BTX, San Diego, CA) at 250 V and 500 μs. Immediately after the pulse, the cell suspension was incubated on ice for 10 min, and the cells were resuspended in complete medium for 48 h. The cells were harvested and subjected to western blotting with the indicated antibodies.

### Immunoprecipitation and western blotting

Immunoprecipitation experiments were performed as previously described [22]. Briefly, samples (500 μg of total protein) were incubated with 2 μg primary antibody overnight at 4°C, after which 20 μl of protein A/G-Plus-Agarose (Santa Cruz Biotechnology, CA, USA) was added to the mixture and incubated for 2 h at 4°C. The immunoprecipitated protein complexes were washed one time with lysis buffer and twice with ice-cold PBS. After discarding the supernatant, the antibody-protein complexes were resuspended in 20 μl Laemmli Sample Buffer (Bio-Rad Laboratories, CA, USA) and boiled for 5 min. The entire sample was separated by 10% SDS-PAGE and assayed by protein immunoblotting.

For western blotting, vehicle control and apigenin-treated cells were lysed in Laemmli Sample Buffer. After electrophoresis, the proteins were electrotransfered to PVDF membranes, blotting with antibodies indicated and visualized by SuperSignal West Dura Extended Duration Substrate (PIERCE, Rockford, IL, USA).

### Statistical analysis

ANOVA was employed for comparisons across multiple groups. The mean of the control was compared with the mean of each individual treatment group by Dunnett's test. All statistical analyses were performed with the Prism 5 software (GraphPad Software Inc., San Diego, CA). Significance was set at *p *< 0.05.

## Results

### Apigenin inhibits CK2 kinase activity and induces growth inhibition and cell cycle arrest in MM cells

**Figure 1:**
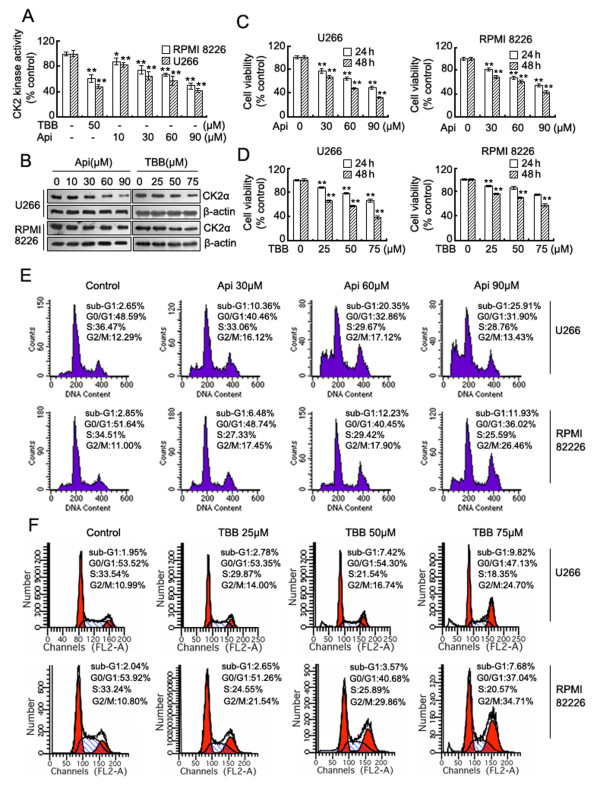
**Apigenin inhibits CK2 kinase activity and induces growth inhibition and cell cycle arrest in MM cells**. (A) CK2 kinase activity was detected in U266 and RPMI 8226 cells exposed to TBB or apigenin for 24 h. Results from 1 of 3 different experiments are shown; data are means ± SD. *Significant difference from control by ANOVA, *p < 0.05, **p < 0.01. (B) CK2α protein levels were detected by western blot analysis in both cell lines treated with different doses of apigenin or TBB for 24 h, and the levels of β-actin serves as the loading control. (C) and (D) U266 and RPMI 8226 cells were grown for 24-48 h in the absence or presence of increasing concentrations of apigenin or TBB respectively. Cell growth inhibition was measured by MTS and is expressed as a percentage of vehicle-treated control; results are presented as means ± SD of three independent experiments. *Significant difference from control by ANOVA, **p < 0.01. (E) and (F) Cell cycle analysis by flow cytometry after apigenin or TBB treatment for 24 h respectively in U266 and RPMI 8226 cells.

Initially, we investigated the effects of apigenin on CK2 kinase activity and expression level and compared these effects with that of TBB, which is a known selective CK2 inhibitor [23]. The results showed that in accordance with TBB, apigenin suppresses CK2 kinase activity (Figure 1A), and reduces CK2α protein levels (Figure 1B) in both U266 and RPMI 8226 cells in a dose-dependent manner. Apigenin and TBB-induced suppression of CK2 was correlated with a dose-dependent decline in MM cell viability (Figure 1C, D), the magnitude of cell proliferation inhibition was greater in U266 cells compared to RPMI 8226 cells. We subsequently evaluated the effect of apigenin and TBB on cell cycle distribution using flow cytometry. Compared to vehicle-only treated controls, the apigenin and TBB treatment resulted in an obvious arrest of cells in G2/M phase after 24 h. The increase in cell number in the G2/M cell population was accompanied by a concomitant decrease in the number in S phase and G0/G1 phases of the cell cycle. Treatment with apigenin led to a dose-dependent accumulation of sub-G1 cells in both U266 and RPMI 8226 cells, thereby indicating that apigenin induces MM cell death, even at relatively low doses (30 μM), whereas TBB only induced minor cell death at 75 μM (Figure 1E, F).

### Apigenin induces apoptosis and downregulates the expression of antiapoptotic proteins in MM cells

**Figure 2:**
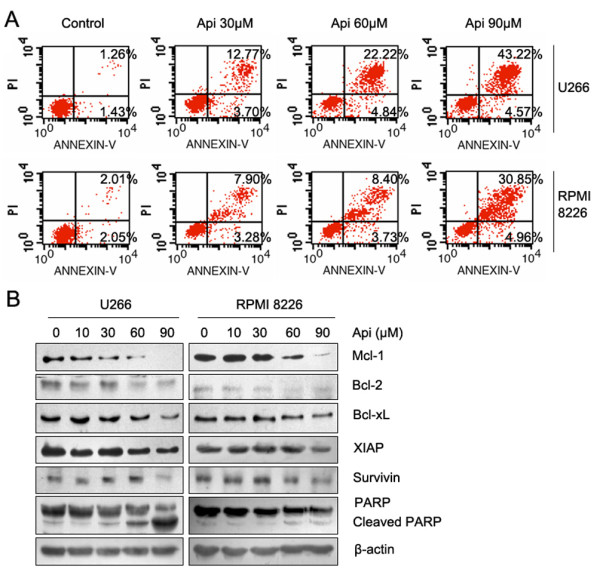
**Apigenin induces apoptosis and downregulates the expression of antiapoptotic proteins in MM cells**. (A) U266 and RPMI 8226 cells were treated with apigenin for 24 h, apoptotic cell death was detected by staining cells with the Annexin V-FLUOS kit and analyzed by flow cytometry. (B) U266 and RPMI 8226 cells were treated with increasing doses of apigenin for 24 h, and the antiapoptotic proteins were detected by western blot analysis using the indicated antibodies; β-actin was used as a loading control.

Next, we treated U266 and RPMI 8226 cells with apigenin for 24 h and analyzed apoptotic cell death using the Annexin V-FLUOS staining Kit. The results revealed a dose-dependent induction of early apoptotic or necrotic/late apoptotic cell death in these two cell lines (Figure 2A). Compared to RPMI 8226 cells, U266 cells showed more cell death, which was consistent with the results of the cell viability assay. Western blot analysis revealed that apigenin caused a dose-dependent decrease in the expression of multiple antiapoptotic proteins, including Mcl-1, Bcl-2, Bcl-xL, XIAP and Survivin. The PARP precursor exhibited a similar reduction, which was accompanied by an increase in the level of its cleaved fragments (Figure 2B). These data indicate that apigenin induced apoptosis in MM cells.

### Apigenin suppresses constitutive and inducible activation of STAT3, AKT, ERK and NF-κB in MM cells

**Figure 3:**
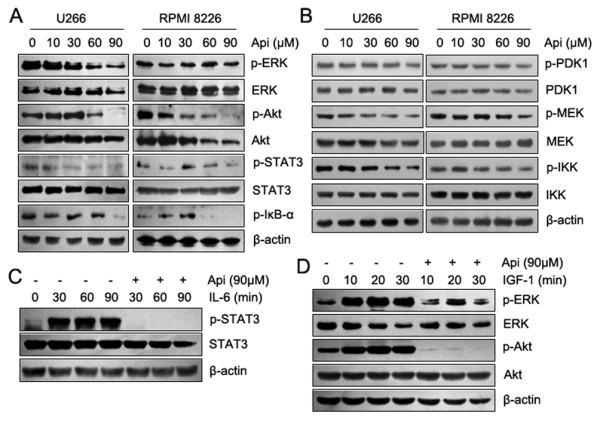
**Apigenin suppresses constitutive and inducible activation of STAT3, AKT and ERK in MM cells**. In (A) and (B), U266 and RPMI 8226 cells were treated with increasing doses of apigenin for 24 h, and the signaling proteins were detected by western blot analysis using the indicated antibodies. In (C) and (D), U266 cells were deprived of serum for 12 h and were treated with rhIL-6 (10 ng/ml) or rhIGF-1 (50 ng/ml) for the indicated times in the presence or absence of apigenin (90 μM). Whole-cell lysates were subjected to western blot analysis using the indicated antibodies.

To investigate further the mechanisms involved in apigenin-induced cell death, we assessed changes in the cellular survival pathways of MM cells. Western blotting results showed that high doses of apigenin decreased the levels of phosphorylated ERK, AKT, STAT3 and IκB-α; the total AKT protein was also decreased (Figure 3A). We also examined the phosphorylation of PDK, MEK and IKK, which are upstream kinase of AKT, ERK and IκB, and found that the phosphorylation levels of these kinases were also reduced to varying degrees (Figure 3B). Unlike RPMI 8226 cells, U266 cells are known to constitutively express IL-6 and the IL-6 receptor, thereby forming an autocrine loop that can sustain autonomous growth [24]. To obtain optimal inhibition of MM proliferation, it is important to block extrinsic signal activation. After a 12 h starvation, we treated U266 cells with IL-6 or IGF-1 in the presence or absence of 90 μM apigenin. As shown in Figure 3B, apigenin completely blocked IL-6-induced activation of STAT3 and IGF-1-induced activation of AKT and partially inhibited IGF-1-induced activation of ERK. These data indicated that apigenin inhibits not only intrinsic cellular survival pathways but also blocks extrinsic cytokine-induced signal transduction.

### Apigenin reduces Cdc37 phosphorylation, disassociates Hsp90/Cdc37/kinase complexes and degrades Hsp90/Cdc37 client proteins

**Figure 4:**
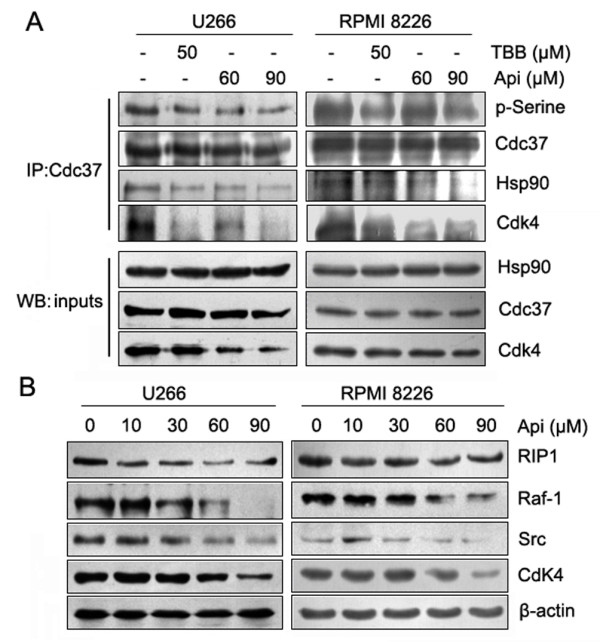
**Apigenin reduces Cdc37 phosphorylation, disassociates Hsp90/Cdc37/kinase complexes and degrades Hsp90/Cdc37 client proteins**. (A) U266 and RPMI 8226 cells were treated with apigenin or TBB for 24 h. Cdc37 protein was immunoprecipitated from whole cell lysates (500 μg each) with an anti-Cdc37 antibody and analyzed by immunoblotting with antibodies against phosphoserine, Hsp90, Cdc37, and Cdk4. For this experiment, 30 μg of the inputs was subjected to western blot to determine the levels of Cdc37, Hsp90, and Cdk4. (B) After treatment of apigenin for 24 h, the protein levels of Cd37 client kinases (RIP1, Raf-1, Src and Cdk4) in U266 and RPMI 8226 cells were detected by western blot analysis.

Previous studies have shown that CK2-mediated Ser13 phosphorylation of Cdc37 is essential for the Cdc37 co-chaperone function involved in recruiting multiple signaling protein kinases to Hsp90 [7,8]. Based on our results reported above, we postulated that apigenin may exert its effect through inhibiting CK2-mediated Cdc37 phosphorylation, and thereby indirectly disrupting Hsp90 chaperone function. To evaluate this hypothesis, we immunoprecipitated Cdc37 and probed blots with anti-phosphoserine, anti-Hsp90, and anti-Cdk4 antibodies to evaluate the phosphorylation of Cdc37 and to detect the association between Cdc37 and its client proteins. Cells were treated with apigenin or TBB (positive control). As shown in Figure 4A, apigenin and TBB decreased the phosphorylation of Cdc37, and the binding between Cdc37 and Hsp90 or its client, Cdk4, indicating that the Hsp90/Cdc37/Cdk4 chaperone complex had been disassociated. To further confirm the effect of apigenin on the Hsp90/Cdc37 chaperone function, additional client proteins were assessed by western blot analysis. The results showed that apigenin induced a dose-dependent degradation of RIP1, Raf-1, Src and Cdk4 kinases (Figure 4B).

### Apigenin-induced proteasome-dependent degradation of Hsp90/Cdc37 client proteins is correlated with inhibition of CK2

**Figure 5:**
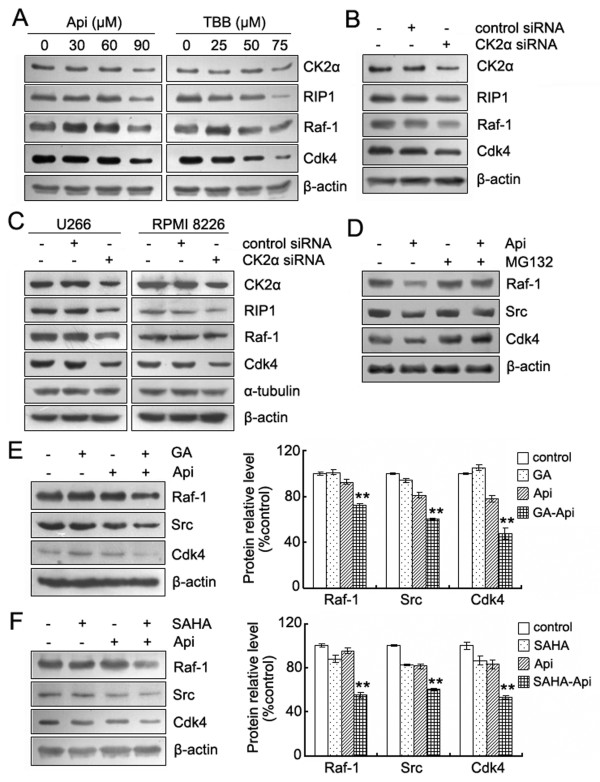
**Apigenin-induced proteasome-dependent degradation of Hsp90/Cdc37 client proteins are correlated with inhibition of CK2**. (A) HeLa cells were treated with the indicated concentrations of apigenin or TBB for 24 h. The protein levels of CK2α, RIP1, Raf-1 and Cdk4 were detected by western blot analysis. (B) HeLa cells were transiently transfected with control siRNA or CK2α siRNA for 48 h. Whole-cell lysates were analyzed by western blotting using the indicated antibodies. (C) Next, siRNA was introduced intoU266 and RPMI 8226 cells using electroporation. After 48 h, the cells were harvested to detect the protein levels by western blot. β-actin, as well as α-tubulin, served as loading control. (D) U266 cells were pretreated with MG132 (1 μM) for 1 h, and the cells were subsequently treated with apigenin (90 μM) for an additional 12 h. Whole-cell lysates were subjected to western blot analysis using antibodies against Raf-1, Src, Cdk4 and β-actin. (E) and (F) U266 cells were incubated with or without the Hsp90 inhibitor GA (0.2 μM) or SAHA (1 μM) for 24 h in the presence or absence of 30 μM apigenin. Whole-cell lysates were subjected to western blotting to determine the levels of Raf-1, Src, Cdk4 and β-actin. The bar graphs on the right show the percentage of intensities of the protein band from each treatment relative to the controls, which were defined as 100%. Values represent the means ± SD. *Significant difference from the three groups was designed by ANOVA, **p < 0.01.

To confirm further that apigenin disrupts the Hsp90/Cdc37 chaperone function via inhibiting CK2; we utilized HeLa cells and compared the effects of apigenin and TBB on CK2α, RIP1, Raf-1 and Cdk4 proteins levels. As depicted in Figure 5A, both apigenin and TBB induced a reduction in CK2α and the degradation of Hsp90Cdc37 client proteins in a dose-dependent manner. These effects are quite similar to those observed in U266 and RPMI8226 cells (Figure 4B). Using siRNA to limit CK2α expression also led to the degradation of RIP1, Raf-1 and Cdk4 proteins in both HeLa cells and the two MM cell lines (Figure 5B, C). In addition, degradation was completely blocked by treatment with the proteasome inhibitor MG132, indicating that the proteasome system was responsible for the apigenin-induced client protein degradation (Figure 5D).

Recent studies have shown that treatment with Cdc37 siRNA compromised the maturation of Hsp90/Cdc37 clients, mediated an increased loss of proteins required for growth and survival and enhanced the sensitivity of cancer cells to Hsp90 inhibitors [14,15]. We examined whether the apigenin-mediated inhibition of the Cdc37 chaperone function might have similar effects when coupled with reagents that affected Hsp90 function. We treated U266 cells with 30 μM apigenin alone or in combination with 0.2 μM geldanamycin (GA), a known Hsp90 inhibitor, or with 1 μM SAHA, which is an HDAC inhibitor that inhibits Hsp90 via enhancing its acetylation [25,26]. All of the reagents were used at levels below their cytotoxic concentrations. The result showed that the combination of apigenin with GA or SAHA had greater effects on depletion of Hsp90/Cdc37 client proteins. Figure 5E and 5F shows that 0.2 μM GA or 1 μM SAHA can enhance the ability of apigenin to deplete the Cdc37 client kinases, Raf-1, Src and Cdk4.

### Apigenin inhibits proliferation, suppresses CK2 activity and depletes Cdc37 client kinases in CD138^+ ^cells from patients with MM

**Figure 6:**
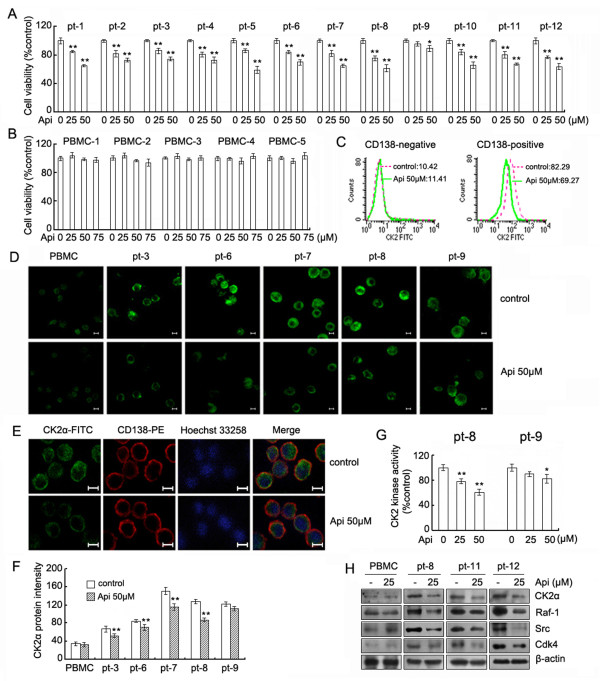
**Apigenin inhibits proliferation, down-regulates CK2 activity, and depletes Hsp90/Cdc37 client proteins in primary MM cells**. (A) Enriched CD138^+ ^cells from 12 multiple myeloma patients were prepared by immunomagnetic bead selection. After treatment of apigenin for 24 h, cell viability was measured by the MTS assay as described in Material and Methods. Values represent the mean ± SD. *Significant difference from control. *p < 0.05, **p < 0.01. (B) PBMCs were treated with the indicated concentrations of apigenin for 24 h and the cell viability was measured by MTS assays. (C) After treatment with apigenin for 24 h, the expression of CK2α in CD138 negative and positive cells was analyzed by flow cytometry and quantified using the mean fluorescence intensity. (D) After treatment with or without 50 μM apigenin for 24 h, PBMCs and the enriched primary myeloma cells were examined by immunofluorescence microscopy to detect the levels of CK2α. The bar is equal to 5 μm. (E) The CD138^+ ^cells were treated with apigenin for 24 h and were stained for CK2α (green), CD138 antigen (red) and DNA (blue). The bar, 5 μm. (F) The fluorescence intensity of each sample from (D) was analyzed using the softWoRx Explorer software. The average pixel intensities from 30 cells of three different fields in each group were measured, and background pixel intensities were subtracted. Values represent the mean ± SD of at least 3 measurements. * Significant difference from control cells, **p < 0.01. (G) After treatment of apigenin for 24 h, the CK2 kinase activity in primary MM cell purified from patient No. 8 and No. 9 was analyzed. *Significant difference from control cells, *p < 0.05, **p < 0.01. (H) Purified PBMC or enriched CD138^+ ^cells from MM patients were treated with apigenin for 24 h, and the whole-cell lysates were subjected to western blotting to determine the levels of CK2α, Raf-1, Src, Cdk4 and β-actin.

The results reported above demonstrate that apigenin has a potent ability to suppress CK2 activity, inhibit Hsp90/Cdc37 chaperone function and induce growth inhibition and apoptosis in MM cell lines. Next, we investigated the effects of apigenin on proliferation of CD138^+ ^cells from 12 patients with MM (Additional file 1) and normal peripheral blood mononuclear cells (PBMCs) from 5 healthy donors. CD138^+ ^cells and PBMCs were exposed to different concentrations of apigenin for 24 h and were examined for cell viability by the MTS assay. The results showed that the CD138^+ ^cells from 11 of the patients with MM were sensitive to apigenin and exhibited a dose-dependent decrease in cellular viability. Cells from one patient (No. 9) showed a slight growth inhibition (Figure 6A). All PBMCs samples were resistant to apigenin, even at higher concentrations (Figure 6B). Next, we determined whether the inhibitory effects of apigenin on proliferation of CD138^+ ^were correlated with CK2 suppression. CD138^+ ^and CD138^- ^cells from MM patients were treated with 50 μM apigenin for 24h, stained and CK2α protein was detected by flow cytometry. As shown in Figure 6C, CD138^- ^cells with low CK2α expression remained unchanged, whereas CD138^+ ^cells with high CK2α expression decreased obviously after apigenin treatment. We also detected the change in CK2α expression by confocal microscopy. Following apigenin exposure for 24 h, 4 out of 5 patients showed various degree of decreased staining for CK2α in CD138^+ ^cells. Staining of CD138^+ ^cells from patient No. 9 was slightly decreased, whereas the staining of PBMC samples was unchanged (Figure 6D), which is consistent with a previous report [6]. We also used CD138 and CK2α or α-tubulin and CK2α double staining to confirm that the decline of CK2α staining was specific. As shown in Figure 6E, apigenin only induced a reduction in CK2α staining, but did not affect the staining of CD138 or α-tubulin (data not shown). The fluorescence intensity of each sample following apigenin treatment was analyzed by the softWoRx explorer software and the changes in CK2α staining in each sample are shown in Figure 6F. To further confirm that the apigenin-induced inhibitory effect of CD138^+ ^MM cells was correlated with suppression of CK2, CD138^+ ^cells from patient No. 8 and No. 9 were further analyzed for CK2 kinase activity. As shown in Figure 6G, apigenin treatment inhibited CK2 activity to a greater extent in CD138^+ ^cells from patient No. 8 than in cells from patient No. 9. Taken together, these results showed that the apigenin-induced decrease in CK2α staining correlated with the decrease in CK2 kinase activity in different samples. Western blot analysis further demonstrated that apigenin induced a decrease in the CK2α and Cdc37 client proteins Raf-1, Src and Cdk4 in CD138^+ ^cells that was similar to the reduction observed in MM cell lines (Figure 6H).

## Discussion

In this study we have shown that a natural dietary flavonoid, apigenin, inhibited the proliferation of MM cell lines and primary MM cells, arrested cell cycle progression, and induced programmed cell death. We demonstrated that apigenin inhibited CK2 activity, thereby leading to inactivation of multiple kinases, including the constitutive and inducible STAT3, AKT, ERK, IκB and their upstream kinase partners PDK, MEK and IKK. Apigenin also downregulated antiapoptotic Bcl-2 family proteins and IAP proteins. We have also shown that the inhibition of CK2-mediated Cdc37 phosphorylation disrupted the Hsp90/Cdc37 chaperone function and led to the degradation of multiple Hsp90/Cdc37 client proteins via the proteasome pathway, which may be the primary mechanism mediating the anticancer activities of apigenin.

Although it is known that apigenin has a selective inhibitory effect on CK2, it has not known if apigenin kills cancer cells through its capacity to interfere with Cdc37 phosphorylation and to disrupt Hsp90 chaperone function. As had been previously reported [6], we observed that primary MM cells and all MM cell lines express constitutively activated CK2. We found that treatment with apigenin downregulated kinase activity in both MM cell lines and the primary MM cells, confirming the suppression of CK2 (Figure 1, Figure 6). In MM cells, the ability of apigenin to inhibit cell proliferation and to induce cell death correlated with its ability to inhibit CK2 activity. It was previously reported that highly CK2α-positive leukemia cells are more sensitive to apigenin-induced cell death than are CK2α leukemia cells with relatively low levels of CK2α [21]. However, in this study, we observed that the sensitivity of MM cells to apigenin-induced cell death depended on whether apigenin effectively inhibited CK2 kinase activity, decreased CK2α protein levels, decreased the phosphorylation of Cdc37 and induced the degradation of Hsp90/Cdc37 client kinases. Consistent with these observations, one of the primary MM cell samples in our analysis (No. 9) exhibited high CK2α expression but had low sensitivity to apigenin, whereas the CK2α low U266 cells were more sensitive to apigenin than CK2α high RPMI 8226 cells. We are currently investigating possible explanations for the failure of apigenin to suppress CK2 activity in particular MM cells.

Importantly, apigenin did not inhibit CK2 activity or exhibit any cytotoxic effects in PBMCs (Figure 6). Apigenin-mediated suppression of CK2 activity was accompanied by reduced phosphorylation of Cdc37 in MM cells, leading to the disassociation of Hsp90/Cdc37/client protein complexes and inducing the degradation of client kinase proteins including RIP1, Raf-1, Src, Cdk4, and AKT via the ubiquitin-proteasome pathway (Figure 4, 5, 6). Since some kinases, such as RIP1, Raf-1 and Src, locate at the upstream of various signal pathways, the degradation of these kinase proteins could lead to the abrogation of their downstream pathways. These findings help to explain how apigenin can inhibit many signaling pathways. In addition to apigenin, resveratrol and epigallocatechin-3-gallate (EGCG) have been reported to induce apoptosis by significantly downregulating CK2 activity in both ALVA-41 and PC-3 prostate cancer cells [27]. Bioactive polyphenolic and flavonoid compounds have demonstrated potential in cancer therapy and cancer chemoprevention, and further studies are needed to determine if CK2 is the common target of these compounds. The possibility that Cdc37 is a secondary target also requires further assessment.

Among the kinases affected by apigenin treatment, receptor interacting protein 1 (RIP1) is of special interest. It has not been determined if RIP1 is a Cdc37 client kinase, but it has been shown that the stability of RIP1 is dependent on Hsp90 chaperone function [28]. Recent studies have demonstrated that RIP1 kinase is a key protein in the cellular decision of cells to live or die upon exposure to different stress signals [29]. Depending on the cellular context and stimulation, RIP1 kinase may participate in three different signal complexes, which have various functions with respect to mediating the activation of NF-κB, apoptosis, or necroptosis [30,31]. Recent studies have reported that apigenin functions as either a pro-apoptotic or anti-apoptotic mediator via suppression of NF-κB activation in malignant cells, such as in pancreatic cancer cells [32] and in various models of inflammation including T cell resistance to activation-induced cell death [33], lipopolysaccharide-stimulated monocytes and macrophages [34], and pancreatic beta-cells [35]. Depletion of the RIP1 protein may be an important mechanism by which apigenin inhibits NF-κB activation to mediate various functions.

The resistance of MM cells to apoptosis involves high expression of members of the Bcl-2 family. These antiapoptotic proteins protect against permeabilization of the mitochondrial outer membrane. The combined total level of Bcl-2, Bcl-xL, and Mcl-1 in the outer membrane determines the resistance of cells to apoptosis [36,37]. In this work, we have shown that apigenin can downregulate multiple antiapoptotic proteins, including Mcl-1, XIAP, Survivin, Bcl-2 and Bcl-xl (Figure 2). Compared with other antiapoptotic proteins, Mcl-1 plays a more important role in the aberrant survival of MM cells [37,38]. As an antiapoptotic protein, Mcl-1 functions either by sequestering Bak on the outer mitochondrial membrane or by heterodimerizing with activated BH3-only proteins including tBid, PUMA, and Bim [39]. Normally, Mcl-1 is constitutively expressed in many MM cells [40,41]. Various extra-cellular stimuli, including interleukins, growth factors, 12-O-tetradecanoyl-phorbol 13-acetate (TPA) and IFN, can upregulate Mcl-1 expression via activation through different signaling pathways [42,43]. Previous studies have shown that down-regulation of Mcl-1 by antisense oligonucleotides is sufficient to induce apoptosis in MM cells and to enhance cancer cell sensitivity to TRAIL, suggesting that Mcl-1 might be a potential therapeutic target for the treatment of several human malignancies, including MM [37,44]. In MM, tumor cells accumulate within the bone marrow by binding to the extracellular matrix proteins and bone marrow stromal cells (BMSCs). The interaction between MM cells and BMSCs induces secretions of various interleukins and growth factors by both cells to promote MM development. Among these interleukins is IL-6, which then triggers VEGF secretion [45]. Although IL-6 and VEGF activate multiple signaling pathways, including Jak-STAT3, ERK and PI3K/AKT, the upregulation of Mcl-1 expression is their main mechanism of mediating survival and proliferation in MM cells [46]. Ideally, the IL-6/VEGF loop ideally supports MM cell growth within the BM microenvironment. A previous study has shown that apigenin can inhibit the expression of VEGF [47]. In the current study, we have demonstrated that apigenin not only suppresses constitutively activated STAT3, ERK, AKT and NF-κB, but it also blocks exogenous IL-6-induced activation of STAT3, and inhibits IGF-1-induced activation of AKT and ERK. These survival signals are important for initiating transcription of Mcl-1 and other antiapoptotic proteins and for maintaining their stability [42]. The inhibitory effect of apigenin may be indirect, as many upstream kinases, such as MEK and IKK, were inactivated as well. The ability of apigenin to suppress constitutive and inducible signaling pathways and to downregulate Mcl-1 also contributes to its cytotoxicity in MM cells.

## Conclusion

Apigenin exhibited anticancer activity against MM cells in vitro. Apigenin decreased Cdc37 phosphorylation by inhibiting CK2 kinase activity, thereby resulting in the disassociation of Hsp90/Cdc37/client complexes and the degradation of Hsp90 client kinase proteins. The depletion of kinases leads to suppression of multiple constitutive and inducible signaling pathways, downregulation of Mcl-1 and induction of apoptosis.

## List of abbreviations

**AML**: acute myeloid leukemia; **BM**: bone marrow; **Cdk4**: Cyclin-dependent kinase 4; **CK2**: Casein kinase 2; **Hsp90**: Heat shock protein 90; **Mcl-1**: Myeloid cell leukemia-1; **MM**: Multiple myeloma; **MTS**: 3-(4,5-dimethylthiazol-2-yl)-5-(3-carboxymethoxyphenyl)-2-(4-sulfophenyl)- 2H-tetrazolium. **NF-κB**: Nuclear Factor κB; **PARP**: poly (ADP-ribose) polymerase; **PBMC**: peripheral blood mononuclear cell; **RIP1**: receptor interacting protein 1; **siRNA**: small interfering RNA; **TBB**: Tetrabromobenzotriazole; **VEGF**: Vascular endothelial growth factor

## Competing interests

The authors declare that they have no competing interests.

## Authors' contributions

MZ carried out the kinase activity assay, Immunoprecipitation and western blotting, participated in analysis and interpretation of data, and helped to draft the manuscript. JM participated in the design of the study and helped to draft the manuscript. HYZ collected the clinical samples and purified the primary MM cells. XHZ participated in the immunofluorescence analysis. ZYD carried out the cytotoxicity assay. YJX carried out the cell cycle and apoptosis assays. XDY designed the study, participated in analysis and interpretation of data, and drafted the manuscript. All of the authors read and approved the final version of this manuscript.

## Supplementary Material

Additional file 1 **Clinical features of patients with MM**. Table indicting the clinical features (age, sex, paraprotein type, and stage) of patients with MM, from which bone marrow samples were obtained. Click here for file
