# Supplementary material for: Apigenin inhibits proliferation and induces apoptosis in human multiple myeloma cells through targeting the trinity of CK2, Cdc37 and Hsp90
Source: Mol Cancer. 2011 Aug 29;10:104. doi: 10.1186/1476-4598-10-104 (PMC3170639; doi:10.1186/1476-4598-10-104)
Supplement: Additional file 1 — Clinical features of patients with MM. Table indicting the clinical features (age, sex, paraprotein type, and stage) of patients with MM, from which bone marrow samples were obtained. [file 1476-4598-10-104-S1.DOC]

Table 1. Clinical features of patients with MM

F, female; M, Male;

| Patient No. | Age | Sex | Paraprotein type | Stage* |
| --- | --- | --- | --- | --- |
| 1 | 77 | F | IgG λ | II |
| 2 | 55 | M | Light λ | IIIA |
| 3 | 35 | F | IgG λ | II |
| 4 | 77 | F | IgA κ | II |
| 5 | 74 | M | IgG λ | II |
| 6 | 39 | M | Light κ | IIIA |
| 7 | 65 | F | IgG κ | IIIA |
| 8 | 47 | M | Light κ | II |
| 9 | 35 | F | IgG λ | I |
| 10 | 55 | M | IgG κ | II |
| 11 | 58 | M | IgG κ | IIIA |
| 12 | 76 | M | IgG κ | II |

*Stage was determined according to the Durie-Salmon staging system.
